# Use of Cone Beam Computed Tomography for Identification of a Distant Causative Tooth: An Unusual Case of an Apical Lesion from a Maxillary Premolar Mimicking That from Maxillary Incisors

**DOI:** 10.1155/2020/8830524

**Published:** 2020-12-30

**Authors:** Shu Abe, Takashi Muramatsu

**Affiliations:** ^1^Heiwa Dental Clinic, Tokyo, Japan; ^2^Department of Operative Dentistry, Cariology and Pulp Biology, Tokyo Dental College, Tokyo, Japan

## Abstract

The article describes an unusual case of an apical lesion at the first premolar with spontaneous pain and swelling around the root and tooth mobility at the maxillary right central and lateral incisors. The patient was a 45-year-old man with a chief complaint of discomfort at the maxillary right central and lateral incisors for one year. Oral examination showed spontaneous pain, swelling, and tooth mobility of the maxillary right central and lateral incisors. Intraoral and panoramic radiographs showed clear apical radiolucency at these sites, although there were no carious incisors. Neither tooth had periodontal pockets, and there was no bleeding on probing sites. A cold thermal examination revealed normal responses of the vital dental pulp. Initial panoramic radiography showed an apical lesion of the maxillary right first premolar, which was root filled and without inflammatory symptoms. A cone beam computed tomography (CBCT) revealed that the apical lesion of the premolar had extensively spread to the anterior through the palatal side, circumventing the palatal side of the adjacent canine, and reached the apical areas of the central and lateral incisors. We treated the apical lesion of the first premolar, and the radiolucent regions had almost disappeared after three years with regard to not only the apical lesion in the first premolar and incisors but also the primary symptoms on the incisors without endodontic treatment. This case shows that CBCT is an effective alternative that allows appropriate treatment to be selected more reliably.

## 1. Introduction

Intraoral periapical radiography continues to be the first-choice examination for endodontic diagnosis, providing information on the presence or absence of apical lesions, their locations, and the anatomical structures of the root canal system and adjacent teeth [1, 2]. However, only two-dimensional information is obtained from intraoral periapical radiographs, and accurate three-dimensional information cannot be obtained even with methodologies such as eccentric projection. Furthermore, considering the difficulty of retaking the same site, the therapeutic effect or healing degree of the actual lesion may not be adequately assessed [3]. Thus, three-dimensional analysis is considered necessary, particularly for the assessment of root resorption and surgical endodontic treatment [3]. The effectiveness of cone beam computed tomography (CBCT) for endodontic diagnosis has been reported [4–6]. We recently reported a case in which apical lesions were not detected using intraoral X-rays but were found on CBCT images [7]. Furthermore, compared with intraoral periapical radiographs, CBCT is an effective examination for the diagnosis of apical lesions due to its higher sensitivity in detecting the lesions [8–12].

We here describe an unusual case of anterior teeth with spontaneous pain caused by an apical lesion from a distant asymptomatic premolar, which was resolved with the disappearance of all symptoms by treating the premolar alone without removal of the dental pulp in the symptomatic teeth.

## 2. Case Report

**Figure 1:**
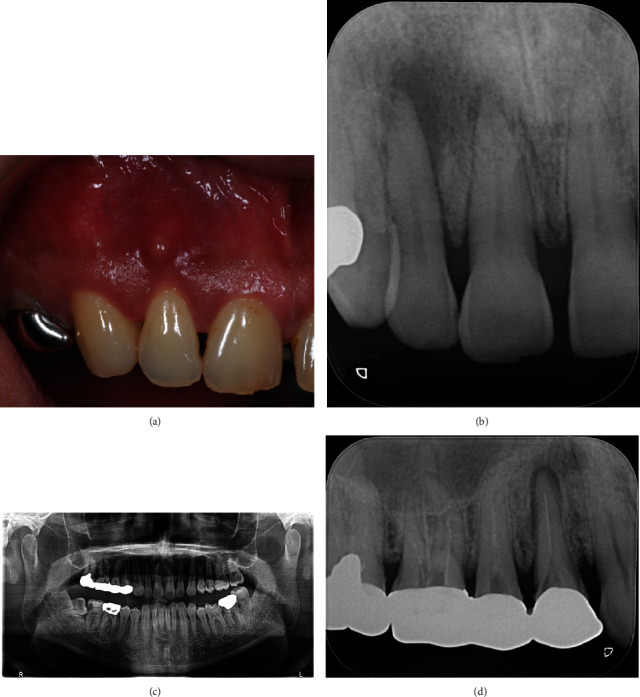
Preoperative photograph and radiographs. Intraoral photographs of the right central and lateral incisor with swelling and spontaneous pain (a). The periodontal pockets were around 1–2 mm circumferentially, and there was no bleeding on probing sites. Intraoral radiographs showed no carious lesions in either incisor, but radiolucent regions were seen around the apexes of the maxillary right central and lateral incisors (b). Both incisors showed normal responses to the dental pulp cold stimulus test. Panoramic radiographs showed numerous apical and carious lesions in the mouth (c). The closest lesion to the site was the maxillary right first premolar. Intraoral periapical radiographs showed apical lesions of the maxillary right first premolar (d).

**Figure 2:**
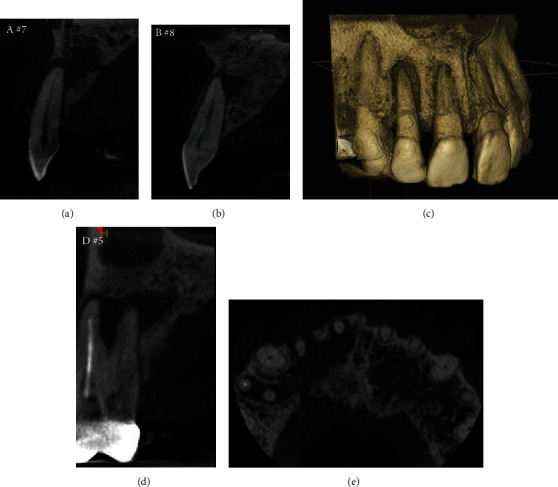
Preoperative CBCT images. Hypodense regions were seen around the apexes of the central and lateral incisors (a, b). Marked apical bone resorption around the lateral incisor (a, c). The palatal root canal of the first premolar was untreated and an apical lesion was observed (d). The apical lesion of the maxillary right first premolar had extensively spread in the anterior direction from the palatal side, destroyed the bone by circumventing the palatal side of the adjacent canine, and reached the apical areas of central and lateral incisors (e).

**Figure 3:**
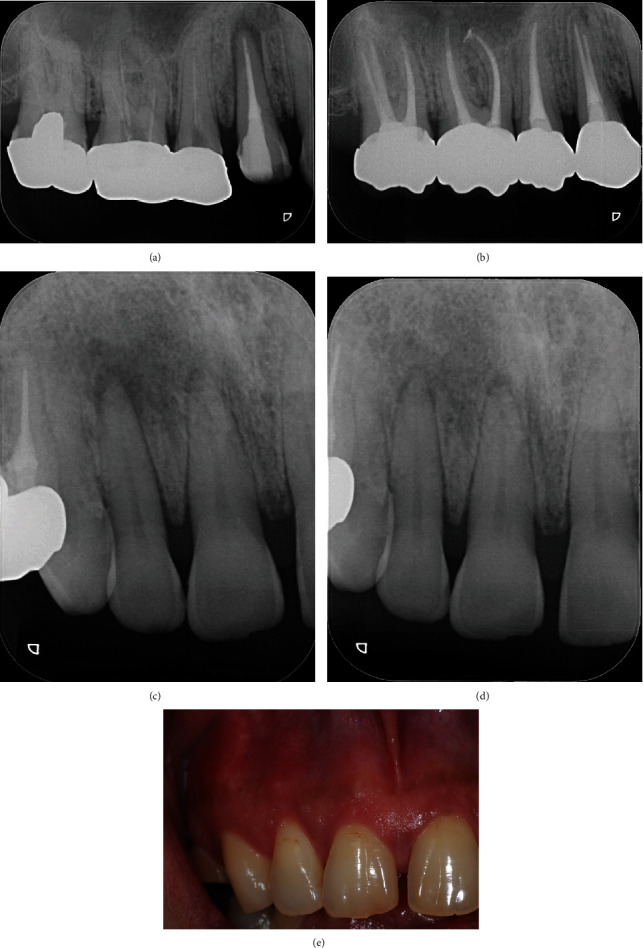
Postoperative intraoral radiographs and photograph. Intraoral radiograph of the maxillary first premolar just after root canal filling (a). Intraoral periapical radiograph of the maxillary right central and lateral incisors after 6-month period from the retreatment of only the first premolar; the radiolucent regions tended to heal (c). Intraoral periapical radiograph of the maxillary first premolar and incisors after a three-year period: the radiolucent region of the premolar was healing. The retreatment of the root canal of the second premolar and first and second molars were realized (b), and the radiolucent regions of incisors had almost disappeared (d). Intraoral photograph of right central and lateral incisor after three years (e).

**Figure 4:**
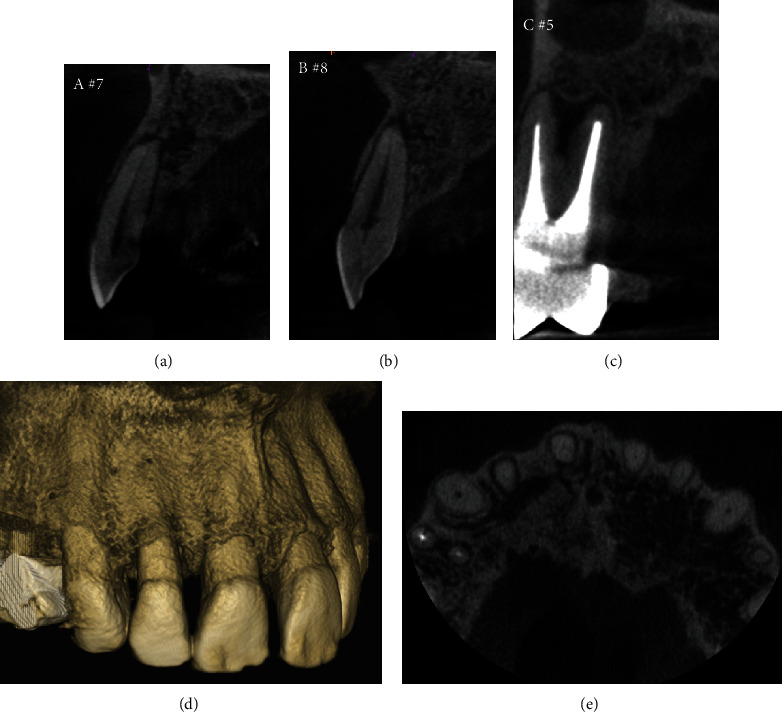
Postoperative CBCT images. CBCT images of the maxillary right central and lateral incisors after a three-year period from the retreatment of only the first premolar; the hypodense regions had almost disappeared (a–c). Volume rendering image after three years showed that the buccal cortical bone had clearly recovered (d). Axial image of maxillary front to premolar area after three years shows the progress of osteoanagenesis in the palatal area (e).

The patient was a 45-year-old man with a chief complaint of discomfort in the maxillary right central and lateral incisors for one year. Mucosal swelling and spontaneous pain were seen at the labial gingiva of the maxillary right lateral incisor, and tooth mobility started at three months before the initial examination (Figure 1(a)). Oral examination revealed the mobility of the maxillary right lateral incisor as grade 2 and that of the central incisor as grade 1. In addition, the patient had both spontaneous and occlusal pain. Intraoral periapical radiographs showed radiolucency at the apical regions of the central and lateral incisors, although no dental caries were detected in these incisors (Figure 1(b)). Both teeth had periodontal pockets around 1–2 mm circumferentially in depth, and there was no bleeding on probing sites. Furthermore, the cold thermal examination revealed normal responses of the vital dental pulp at both incisors. Panoramic radiography showed numerous dental caries and apical lesions in the oral cavity (Figure 1(c)). The closest lesion to the site of the chief complaint was detected at the maxillary right first premolar, which was previously treated by root canal treatment. A connecting bridge was attached, linking from the first premolar to the first molar, and there was no occlusal pain on percussion. The adjacent maxillary right canine was intact without caries or periodontal disease. Intraoral periapical radiography revealed an apical lesion in the maxillary right first premolar (Figure 1(d)). However, the apical lesion appeared small on intraoral periapical radiographs, there was no swelling of the mucosa around the root, and the patient had no symptoms. CBCT was performed using Trophypan Smart Osiris 3D (Carestream Dental, Atlanta, GA) to obtain three-dimensional information. The CBCT images revealed marked apical radiolucency and bone resorption around the lateral incisor. We also found apical radiolucency with destruction of the cortical bone on the buccal side, measuring ≥12 mm in diameter on the lateral incisor, and an apical radiolucency measuring ≥8 mm in diameter on the central incisor (Figures 2(a)–2(c)). Root canal filling material was found on the buccal root of the maxillary right first premolar, and no root canal filling agents were seen in the palatal root, with an apical lesion measuring more than 10 mm in diameter on the palatal side (Figure 2(d)). Furthermore, the apical lesion had spread extensively from the palatal apical lesion and reached the apical areas of the central and lateral incisors that were the sites of the chief complaint (Figure 2(e)). Based on these results, the clinical diagnostic was that the apical radiolucency of the chief complaint was derived from the apical lesion of the palatal root at the maxillary right first premolar. Therefore, we planned to retreat the root canal of the first premolar, which was considered as the causative tooth. To perform endodontic caries treatment, the connective bridge on the maxillary right molars was cut between the first and second premolars to remove the crown on the first premolar alone. Subsequently, we used a rubber dam and removed the gutta percha in the buccal roots using ProTaper Retreatment files D1 and D2 (Dentsply Sirona Endodontics, Ballaigues, Switzerland) and a #10 K-file (Dentsply Sirona Endodontics) to locate and measure the working length of the buccal and palatal root canals with an electronic apex locator (Root ZX; J. Morita Mfg. Corp., Kyoto, Japan). WaveOne Gold (Dentsply Sirona Endodontics) was then used to expand the root canal to have a large tip size. We used 6% sodium hypochlorite for all intermediary procedures. After root canal enlargement, we used 17% ethylenediaminetetraacetic acid (EDTA) for 1 min and ultrasound with an EndoActivator (Dentsply Sirona Endodontics) to irrigate the root canal wall dentin. The fluid inside the root canal was then aspirated and dried with a paper point, and calcium hydroxide (Fujifilm Wako Pure Chemical Corporation, Osaka, Japan) mixed with sterilized water was applied and given a temporary hydraulic seal with Caviton EX (GC Corp., Tokyo, Japan). On the second visit (four weeks later), root canal filling was performed using a resin sealer (AH Plus; Dentsply Sirona Endodontics) with the core carrier method (Gutta Core, Dentsply Sirona Endodontics). Abutment was prepared using a flowable resin composite, and a temporary crown was placed (Figure 3(a)). This treatment led to the disappearance of swelling at the maxillary right central and lateral incisors approximately two weeks postoperatively, and intraoral periapical radiographs showed a tendency of disappearance of the apical radiolucency approximately six months postoperatively (Figure 3(c)). After three years, intraoral periapical radiographs and CBCT images showed that apical radiolucency almost disappeared around the maxillary first premolar and incisors (Figures 3(b), 3(d), 4(a), 4(b), and 4(c)). The mobility of the central and lateral incisors improved to grade 0, and the dental pulp was preserved, leaving vital teeth with no symptoms (Figure 3(e)).

The apical radiolucencies of the maxillary right central and lateral incisors, the sites of the chief complaint in this patient, were large and required approximately three years until clear osteoanagenesis was achieved. CBCT shows bone destruction around the lateral incisor, also visible on the periapical radiography, and there is also a hypodense image between the roots of the premolar (Figure 3(d), 4(a), and 4(e)), requiring a longer follow-up. Swelling of the mucosa diminished in approximately two weeks, but it took approximately one-and-a-half years for the patient's discomfort to completely disappear.

## 3. Discussion

In this case, the patient exhibited spontaneous pain and swelling around the roots and tooth mobility of the maxillary right central and lateral incisors, and clear apical radiolucency was seen on panoramic and intraoral radiographs. However, these examinations did not identify a definite cause of the apical radiolucencies, and we, therefore, performed CBCT. We finally identified the distant causative tooth and obtained good results.

Although the efficacy of CBCT in endodontic treatment has been reported in numerous articles [4, 5, 11], it should not be routinely used to detect apical lesions, owing to the high level of radiation exposure to patients [13], and should only be used when the cause cannot otherwise be identified [1, 2, 14]. Torabinejad et al. [15] reported that CBCT detected apical lesions in ≥20% of patients without apical radiolucency on intraoral periapical radiographs. However, the report also warned clinicians to avoid overtreating cases that do not require treatment, as bone resorption in these apical radiolucencies has not been pathologically diagnosed [15]. In this case, CBCT allowed the identification of a distant causative tooth, and treatment of that tooth alone resolved the apical lesion. Accurate identification of the causative tooth, which was located at a distant site from the site of the chief complaint, would have been difficult with conventional intraoral periapical radiographs alone. Thus, the present case demonstrated that CBCT is an extremely effective examination for exploring the cause of a lesion that cannot be identified by conventional radiography.

In this case, a sinus tract was observed in the anterior maxillary region near the causative tooth. Regarding the spread of drainage pathways from periapical abscesses, bone thickness may be a factor [16]. Porto et al. [17] reported the thickness from the root apex to the labial/buccal and palatal cortical bones of the upper anterior teeth and molars. According to their report, the maxillary cortical bone at the labial/buccal side is thinner than that of the palatal side at the incisor and premolar regions. A sinus tract typically follows the path of least resistance through the alveolar bone [18, 19]. In this case, the maxillary first premolar, as the causative tooth, had thicker bone on the palatal side than on the buccal side and in the anterior direction rather than the molar direction on the palatal side. Therefore, the abscess drainage from the first premolar passed through the palatal side of the canine and ultimately opened at the gingiva between the central and lateral incisors.

In conclusion, CBCT revealed the definite cause of apical lesions of the sinus tract and allowed a precise diagnosis of apical radiolucency when diagnosis by conventional dental radiography proved inadequate.
